# A novel patient-reported outcome instrument assessing the symptoms of paroxysmal nocturnal hemoglobinuria, the PNH-SQ

**DOI:** 10.1186/s41687-021-00376-0

**Published:** 2021-09-28

**Authors:** R. Paola Daly, Jessica J. Jalbert, Shannon Keith, Tara Symonds, Jamile Shammo

**Affiliations:** 1 Clinical Outcomes Solutions, Chicago, IL USA; 2 Regeneron Pharmaceuticals, Inc., 777 Old Saw Mill River Rd, Tarrytown, NY 10591 USA; 3 Clinical Outcomes Solutions, Folkestone, UK; 4 Rush University Medical Center, Chicago, IL USA

**Keywords:** PNH, PRO, QoL, Symptom

## Abstract

**Background:**

Patient-reported outcome measures (PROs) used to measure symptoms of patients with paroxysmal nocturnal hemoglobinuria (PNH) in trials do not measure PNH symptoms comprehensively and do not assess daily fluctuations in symptoms. Following a literature review and consultation with a PNH expert, we drafted the PNH Symptom Questionnaire (PNH-SQ) and a patient-centric conceptual model of PNH symptoms and impacts. We then interviewed 15 patients with PNH to assess comprehensiveness of symptom capture from the patient perspective and to cognitively debrief the PNH-SQ. Patient interview data were also used to finalize the PNH conceptual model.

**Results:**

Participants mentioned 27 signs or symptoms of PNH spontaneously or after being probed; 93% reported experiencing ≥ 1 PNH symptom. Concept saturation was reached for all PNH symptoms. Further, interviews confirmed the instrument captured the most common PNH symptoms, including fatigue (87%), abdominal pain (60%), and difficulty swallowing (47%), with fatigue ranked as the most bothersome symptom. The interviews demonstrated that participants understood the items of the PNH-SQ (90–100%); considered the symptoms relevant (> 50– > 90%); the recall period appropriate (> 80–100%); and the response options suitable (> 80–100%). Participants also suggested changes regarding item redundancy and relevance; this feedback was used to finalize the instrument.

**Conclusions:**

The finalized PNH-SQ assesses the presence and severity of 10 symptoms—abdominal pain, chest discomfort, difficulty sleeping, difficulty swallowing, difficulty thinking clearly, fatigue, headache, muscle weakness, pain in the legs or back, and shortness of breath—over 24 h. The PNH-SQ is a content-valid questionnaire suitable for assessing daily symptom presence and severity in PNH clinical trials.

## Background

Paroxysmal nocturnal hemoglobinuria (PNH) is a rare, acquired, life-threatening disease of the blood caused by a chronic dysregulation of the complement system [1] and is characterized by hemolytic anemia, thrombosis, and impaired bone marrow function [2, 3]. The prevalence of PNH is estimated to be between 10 and 15 per million [4, 5], with a mean age of diagnosis between 30 and 45 years [6]. Patients with PNH experience a high symptom burden that impacts and significantly reduces quality of life [3, 5, 7]. In the International PNH Registry, among the 856 patients for whom symptom data were available, the most commonly reported symptoms were dyspnea (64%), headache (63%), and fatigue (80%) [7]. Patients experiencing symptoms of PNH in the previous 6 months had significantly lower quality of life scores, as measured by the European Organisation for Research and Treatment of Cancer Quality of Life of Cancer Patients (EORTC QLQ-C30), than patients not experiencing symptoms [7]. In addition, in a subset of 506 participants aged 18–59, 17.4% reported that their PNH was the reason they were not working or working part-time instead of full-time [7]. In a study consisting of concept elicitation interviews with 29 patients with PNH, 97% of participants reported experiencing fatigue, 76% experienced headache, 66% experienced dyspnea, and 59% experienced abdominal pain [8]. Over one-half of patients reported symptom severity as moderate to severe and some patients experienced certain symptoms with high frequency; 52% of those with fatigue and 41% of those with headaches reported that their symptoms occurred frequently or almost constantly [8]. The percent of patients who were unemployed due to PNH was similar to that reported in the International PNH Registry (17%) [8].

Given that most patients with PNH experience symptoms that have a substantial impact on quality of life and ability to perform daily activities, symptoms should be carefully measured and tracked in trials evaluating new treatment options for PNH. We conducted a literature review and no fit-for-purpose patient-reported outcome (PRO) measure existed to assess symptoms of PNH in clinical trials. While the EORTC QLC-C30 and Functional Assessment of Chronic Illness Therapy-Fatigue (FACIT-Fatigue) have been used to support labeling for PNH treatments, these measures were not developed specifically for patients with PNH. The EORTC QLQ-C30 has significant gaps in symptom coverage and measures symptoms that are not relevant to patients with PNH (e.g. nausea, vomiting, constipation) [6, 8]. Moreover, both the EORTC-QLQ-C30 and the FACIT-Fatigue assess symptoms over the past week and interviews with patients with PNH suggest that symptom frequency may vary [8].

The objective of this study was to gain a better understanding of the patient experience related to PNH symptoms and to develop a content-valid questionnaire—the PNH Symptom Questionnaire (PNH-SQ)—for use in clinical trials to assess the presence, severity, and day-to-day variations in PNH-specific symptoms.

## Methods

**Fig. 1:**
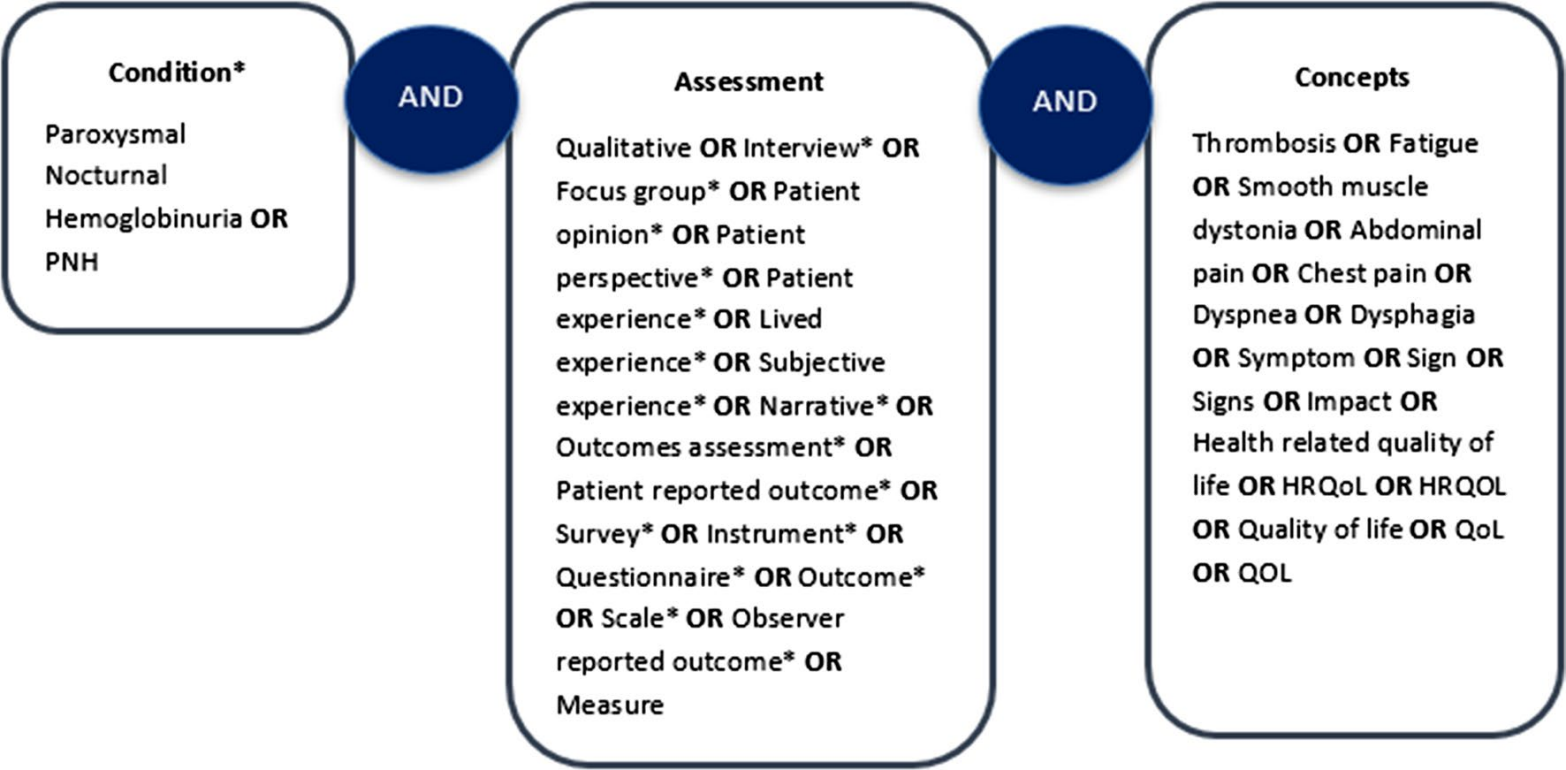
Literature review search terms

The development of the PNH-SQ was conducted in accordance with the Food and Drug Administration (FDA)’s guidance on PRO measure development [9]. The content and design of the PNH-SQ was informed by a review of the empirical literature, review of the content of PRO measures used in registrational trials to assess symptoms of PNH (i.e., the FACIT-Fatigue and the EORTC-QLQ-C30), and, in October 2018, through a discussion with a hematologist with over 20 years of experience treating patients with PNH. The literature search was conducted in OVID across three databases (Medline, PsychInfo, and Embase) using the terms described in Fig. 1 and between the time period 2006–2018. This time period was chosen so that measure development articles published from the issuance of the draft FDA Guidance on PRO development [10] to the year in which the search was conducted would be included. From this search, four qualitative research papers were identified and reviewed [7, 11–13]. Data on symptoms were extracted from each study. The results of the literature review were coupled with findings from the PRO review and clinician discussions to inform the development of a preliminary conceptual model of PNH and the first draft of the questionnaire. A translatability assessment was also conducted by a global translation company, Transperfect, on the PNH-SQ in 14 other languages including Czech, German, Italian, Korean, Polish, Thai, as well as UK and South African English. For this review, a linguistic validation expert reviewed the English version of the PNH-SQ to identify any text or concepts that could be difficult to translate. We then conducted hybrid patient interviews which consisted of concept elicitation (CE) and cognitive debriefing (CD). Patient feedback and discussions with a physician with experience treating patients with PNH (author JS) were then used to finalize the instrument.

### Concept selection and PNH-SQ construction

The selection of key PNH symptoms for inclusion in the PNH-SQ was based on findings from the literature, discussions with a physician treating patients with PNH, and through collaborative efforts among co-authors. The initial draft of the PNH-SQ assessed the presence/absence of 12 symptoms: fatigue, shortness of breath, muscle weakness, headache, abdominal pain, leg/back pain, chest discomfort, sexual difficulties, difficulty sleeping, cognitive problems (i.e., difficulty focusing and difficulty thinking clearly), and difficulty swallowing. If a symptom was reported as present, the participant was then asked to rate symptom severity. The PNH-SQ is intended to be administered daily and has a 24-h recall period.

### Participant recruitment

Interviews with PNH patients were conducted to (1) identify and confirm the important and relevant symptoms from the patient’s perspective (i.e., CE interviews) and (2) to evaluate the respondent’s ability to understand and complete the PNH-SQ (i.e., CD).

To this end, we sought to interview 15 patients with PNH. Given that PNH is a rare disease, making recruitment challenging, a conservative estimate of when concept saturation may be reached was set at 15 participants. Following institutional review board approval, potentially eligible study participants were identified through 2 patient groups in the United States: the Aplastic Anemia and Myelodysplastic Syndromes International Foundation (AA-MDS) and the Rare Patient Voice (RPV). Prior to confirming eligibility, patients were presented with study information and provided written informed consent. To be eligible for the study, patients had to meet the following inclusion criteria: at least 18 years of age, have a clinical diagnosis of PNH (written confirmation by the patient’s physician was sought), not have an active systemic autoimmune disease, not be currently participating in a clinical trial, and not have an active, co-existing chronic anemia unrelated to PNH (e.g., aplastic anemia or myelodysplastic syndrome).

### Patient interviews

**Fig. 2:**
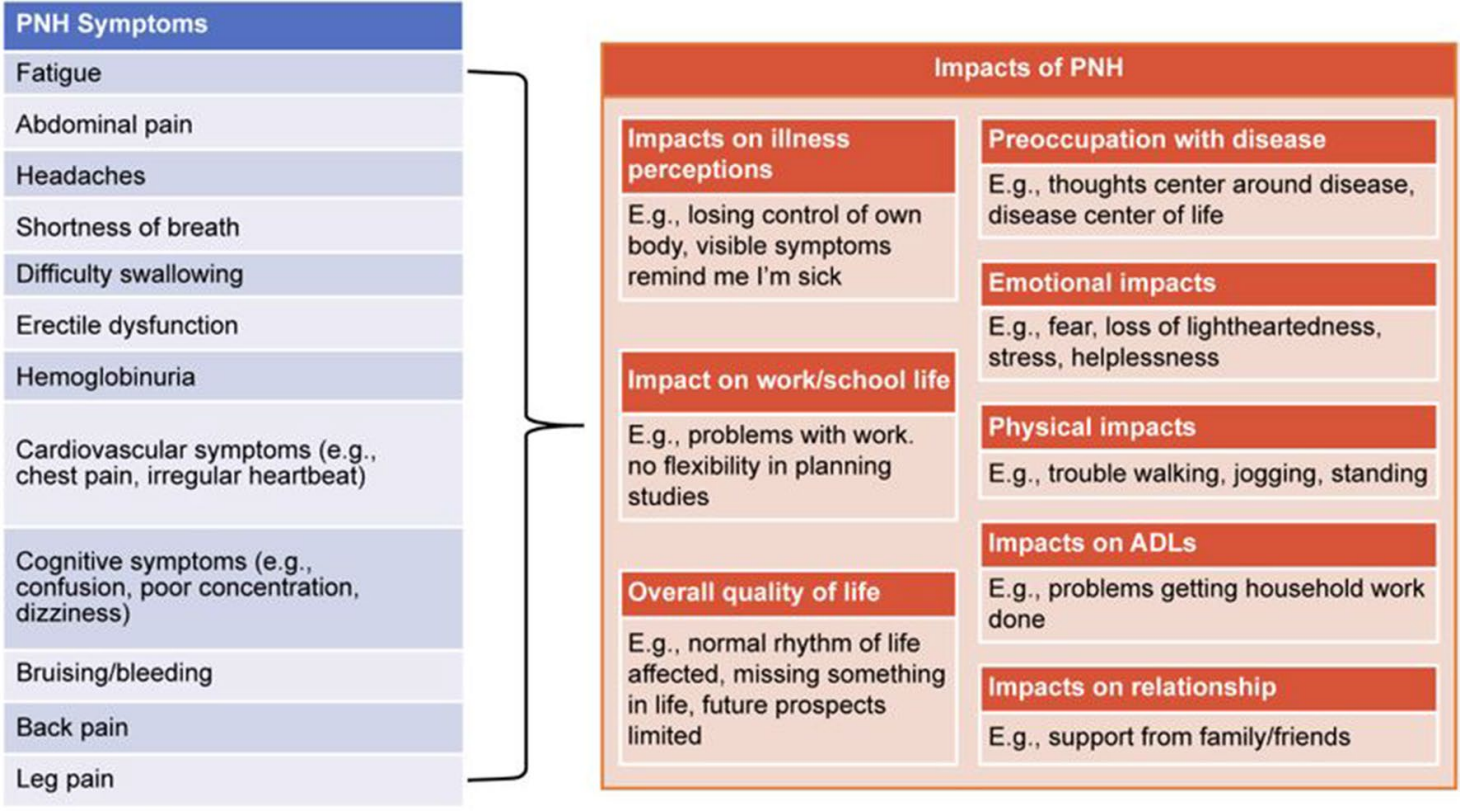
Conceptual model of PNH. *PNH*, paroxysmal nocturnal hemoglobinuria

Interviews, approximately 60 min in duration, were conducted via telephone or in-person. Interview mode was determined by what was most convenient for the participant. Using a semi-structured interview guide, the interviews consisted of 2 distinct parts: (1) CE, to assess the comprehensiveness of symptoms captured by the PNH-SQ and (2) CD, to evaluate the participant’s ability to understand and respond to the PNH-SQ. During the CE portion of the interview, participants were asked open-ended questions about their symptoms and experience living with PNH, providing a full, participant-led picture of symptoms and impacts of PNH. To ensure the symptoms discussed by the participant were believed to be related to disease and not a treatment side effect or other health issue, participants were specifically asked about the symptoms they experienced due to their PNH. To ensure that all relevant symptoms were captured in the PNH-SQ, interviewers were instructed to ask the participant if they experienced symptoms from the PNH-SQ that were not mentioned spontaneously. Concept saturation was assessed by dividing interviews into 3 equal groups of 5 participants based on the order in which the interviews were conducted. Saturation is considered to be achieved if a downward trend is observed in the elicitation of new symptoms (i.e., if few or no new symptoms emerge in the final transcript group). Additionally, the concepts mentioned spontaneously by participants were used to confirm the relevance of the patient-centric conceptual model of PNH (Fig. 2). This model shows the symptoms and impacts patients experience and believe are directly related to their PNH, first gathered by review of qualitative literature [7, 8, 11–13] and then confirmed in the interviews with patients. The conceptual model focuses on disease symptoms and impacts while excluding treatment side effects and related impacts.

During the CD portion of the interview, participants were asked to complete the PNH-SQ. Participants were instructed to use a “think aloud” procedure in which they verbalize their thoughts as they complete the questionnaire. For example, when participants selected a response for symptom severity, they were asked to describe why they selected that response. Data from CD were used to evaluate the relevance of each item and assess participant’s understanding of all instructions, items, recall period, and response options and to confirm the suitability of the recall period and response options. We included 2 items for the cognitive issues concept (i.e., “difficulty thinking clearly” and “difficulty focusing”) with the intent of asking patients if they represented the same concept, and, if so, which item best captured their experience. Following the review of the PNH-SQ with the participant, interviewers also asked patients if they thought any symptoms were missing from the PNH-SQ, and if so, which symptoms they would recommend for inclusion.

### Interview analysis

All interviews were audio-recorded, transcribed, coded, and analyzed using NVivo v12.0. For the CE data, the frequency with which participants reported each symptom concept was tallied. For the CD data, the frequency with which participants reported issues with, or understanding of, item content was tallied. Results were summarized in tabular format.

## Results

Most interviews (9/15) were conducted via telephone, 2 were conducted using telephone with video, and 4 were conducted in-person. The mean age of patients was 42.8 years (SD: 10.4), 53.3% were women, 93.3% were White, average years since diagnosis was 13.4 years (SD: 10.3), and 66.7%, 13.3%, and 20.0% reported that they had very mild/mild, moderate, and severe/very severe disease, respectively (Table 1). Among the 12 patients who were asked about treatment, 7 participants were currently treated with eculizumab. Ultimately, clinical confirmation of a PNH diagnosis was received for 4 participants.

**Table 1:** Characteristics of interview participants (N = 15)

Participant characteristic	Very mild to mild (N = 10)	Moderate (N = 2)	Severe to very severe (N = 3)	Total (N = 15)
Age, years				
Mean (SD)	41.5 (11.10)	52.5 (3.54)	40.7 (9.50)	42.8 (10.42)
Q1	33.0	50.0	31.0	33.0
Median	41.0	52.5	41.0	41.0
Q3	47.0	55.0	50.0	50.0
Min–Max	28–63	50–55	31–50	28–63
Sex, n (%)				
Female	5 (50.0)	1 (50.0)	2 (66.7)	8 (53.3)
Male	5 (50.0)	1 (50.0)	1 (33.3)	7 (46.7)
Race, n (%)				
White	10 (100.0)	2 (100.0)	2 (66.7)	14 (93.3)
Other	0 (0.0)	0 (0.0)	1 (33.3)	1 (6.7)
Ethnicity, n (%)				
Hispanic/Latino	0 (0.0)	0 (0.0)	1 (33.3)	1 (6.7)
Not Hispanic/Latino	10 (100.0)	2 (100.0)	2 (66.7)	14 (93.3)
Education, n (%)				
High school diploma (or GED)	0 (0.0)	0 (0.0)	2 (66.7)	2 (13.3)
Some college or certification program	1 (10.0)	0 (0.0)	0 (0.0)	1 (6.7)
College or university degree	5 (50.0)	2 (100.0)	1 (33.3)	8 (53.3)
Graduate degree	4 (40.0)	0 (0.0)	0 (0.0)	4 (26.7)
Employment status, n (%)				
Other	0 (0.0)	0 (0.0)	1 (33.3)	1 (6.7)
Employed full-time	8 (80.0)	0 (0.0)	1 (33.3)	9 (60.0)
Employed part-time	1 (10.0)	1 (50.0)	0 (0.0)	2 (13.3)
Homemaker	0 (0.0)	0 (0.0)	1 (33.3)	1 (6.7)
Retired	1 (10.0%)	1 (50.0%)	0 (0.0%)	2 (13.3%)
Time since diagnosis, years				
Mean (SD)	11.6 (9.11)	27.0 (1.41)	10.3 (12.10)	13.4 (10.25)
Q1	4.0	26.0	1.0	4.0
Median	10.5	27.0	6.0	12.0
Q3	18.0	28.0	24.0	24.0
Min–Max	0–28	26–28	1–24	0–28
Treatment status, n (%)				
Unknown	3 (30.0)	0 (0.0)	0 (0.0)	3 (20.0)
On treatment	3 (30.0)	2 (100.0)	2 (66.7)	7 (46.7)
Treatment naïve	4 (40.0)	0 (0.0)	1 (33.3)	5 (33.3)

*GED* general educational development, *Q* quarter, *SD* standard deviation

### Concept elicitation

During the interviews, participants mentioned 27 signs or symptoms of PNH either spontaneously or after being probed. Fourteen of 15 (93.3%) participants reported experiencing symptoms due to PNH; 1 female participant reported that she did not experience symptoms in the 18 years since her PNH diagnosis. The most common symptoms mentioned were fatigue (n = 13, all spontaneous), abdominal pain (n = 9, all spontaneous), difficulty swallowing (n = 7, 4 spontaneous), sexual difficulties (n = 6, 2 spontaneous), and back pain (n = 6, 5 spontaneous) (Table 2). Symptoms spontaneously mentioned by at least 5 participants included: fatigue (n = 13), abdominal pain (n = 9), muscle weakness (n = 5), back pain (n = 5), cognitive difficulties (n = 5), and shortness of breath (n = 5). In addition to PNH symptoms, participants also frequently spontaneously mentioned signs of PNH, such as dark urine (n = 11), bruising (n = 4), paleness (n = 3), and yellow eyes (n = 1). When participants were asked which symptom of PNH was most bothersome, the majority (n = 8) considered fatigue to be the most bothersome symptom, followed by cognitive issues (n = 3), and stomach pain (n = 2).

**Table 2:** Signs and symptoms reported during concept elicitation (N = 15)

Symptoms and signs reported	Spontaneous	Probed	Total N	Percent experienced symptom, %
Fatigue*	13	0	13	87
Dark urine	11	0	11	73
Abdominal (stomach) pain*	9	0	9	60
Difficulties swallowing*	4	3	7	47
Sexual difficulties*	2	4	6	40
Back pain*	5	1	6	40
Cognitive difficulties*	5	0	5	33
Headache*	4	1	5	33
Muscle weakness*	5	0	5	33
Shortness of breath*	5	0	5	33
Bruising	4	0	4	27
Sleep problems*	3	1	4	27
Paleness	3	0	3	20
Leg pain*	3	0	3	20
Chest discomfort*	2	0	2	13
Dizziness	2	0	2	13
Fast heart rate	2	0	2	13
Muscle aches	2	0	2	13
Yellow eyes	1	0	1	7
Sore spleen	1	0	1	7
Sensitive nerves	1	0	1	7
Heartburn	1	0	1	7
Hoarse voice	1	0	1	7
Vision problems	1	0	1	7
Urinary spasms	1	0	1	7
Ascites	1	0	1	7
Nausea	0	1	1	7

^*^Denotes an item listed in the Paroxysmal Nocturnal Hemoglobinuria-Symptom Questionnaire (PNH-SQ)

Of the 27 signs and symptoms that were elicited spontaneously or after probing, 66.7% emerged in the first 5 interviews (Table 3). Two concepts, nausea and ascites, emerged in the last set of interviews but were considered by a physician who treats patients with PNH (co-author J.S.) as idiosyncratic to that patient. As such, concept saturation was reached for PNH symptoms with 15 interviews and the conduct of additional interviews was considered unnecessary. Notably, all 12 symptoms included in the first draft of the PNH-SQ were spontaneously mentioned by at least 1 participant during CE (Table 3). The data from CE were also used to finalize the patient-centric conceptual model of PNH symptoms and impacts (Fig. 2), for which no changes were needed.

**Table 3:** Concept saturation analysis

Symptom	Set 1 *n* = 5	Set 2 *n* = 5	Set 3 *n* = 5	Saturation met?
Fatigue	✓	✓	✓	Y
Sleep problems	✓	✖	✓	Y
Abdominal pain	✓	✓	✓	Y
Chest discomfort	✓	✖	✓	Y
Shortness of breath	✓	✓	✓	Y
Muscle weakness	✖	✓*	✓	Y
Cognitive difficulties	✓	✓	✓	Y
Difficulties swallowing	✓	✓	✖	Y
Sexual difficulties	✖	✓*	✖	Y
Yellow eyes	✓	✖	✖	Y
Headache	✓	✓	✖	Y
Sore spleen	✓	✖	✖	Y
Sensitive nerves	✓	✖	✖	Y
Back pain	✓	✓	✓	Y
Dark urine	✓	✓	✓	Y
Paleness	✓	✖	✓	Y
Bruising	✓	✓	✓	Y
Heartburn	✓	✖	✖	Y
Hoarse voice	✖	✓*	✖	Y
Fast heart rate	✖	✓*	✓	Y
Muscle aches	✖	✓*	✓	Y
Urinary spasms	✓	✖	✖	Y
Ascites	✖	✖	✓*	N

✓Concept discussed in interview set

✖ Concept not discussed in interview set

*N* No, *Y* Yes

^*^Represents emergence of a new concept

### Cognitive debrief of the PNH-SQ

#### Item relevance

**Fig. 3:**
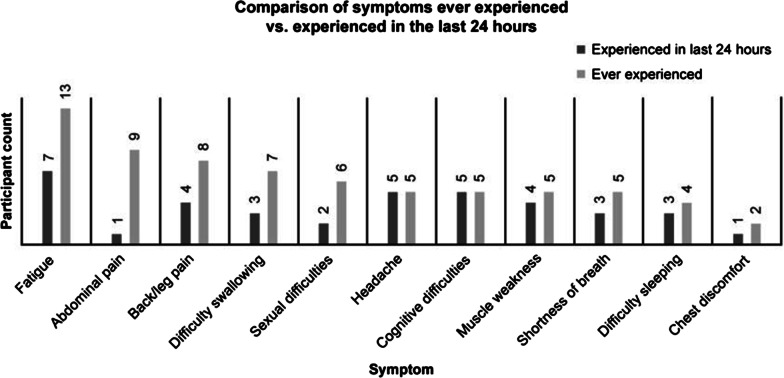
Frequency of PNH symptoms experienced. *PNH* paroxysmal nocturnal hemoglobinuria

Participants completed the PNH-SQ and were then asked about the relevance of each item, regardless of whether they had experienced it in the last 24 h. The majority of study participants found all items in the PNH-SQ relevant (Table 4). Item relevance ranged from 54.5% (for “difficulty sleeping”) to 100% (for “fatigue”). All symptoms on the PNH-SQ had been experienced by at least 1 patient in the last 24 h. At least one-third of participants reported experiencing fatigue (n = 7), headache (n = 5), or cognitive difficulties (n = 5), in the last 24 h (Fig. 3). There appeared to be some variability in how often patients experienced symptoms. For example, while 60% of patients (9/15) experienced abdominal pain due to their PNH in the past, only 1 participant reported having abdominal pain in the past 24 h. In contrast, > 50% of patients reporting having ever experienced fatigue, back pain, headache, cognitive difficulties, muscle weakness, shortness of breath, or difficulty sleeping also reported having experienced the symptom in the past 24 h.

**Table 4:** Relevancy of items of the PNH-SQ

	Relevant	Not relevant	Maybe relevant	Number of participants asked	Percent relevant, %
Fatigue	15	0	0	15	100
Shortness of breath	8	1	4	13	62
Muscle weakness	9	3	1	13	69
Headache	8	4	0	12	67
Abdominal pain	10	2	0	12	83
Pain in back or legs	11	1	0	12	92
Chest discomfort	8	5	0	13	62
Sexual difficulties	9	0	2	11	82
Difficulty sleeping	6	1	4	11	55
Difficulty focusing	11	1	1	13	85
Difficulty thinking clearly	10	2	0	12	83
Difficulty swallowing	10	3	0	13	77

*PNH-SQ* Paroxysmal Nocturnal Hemoglobinuria-Symptom Questionnaire

While an almost equal number of participants felt that both “difficulty focusing” (10/13) and “thinking clearly” (10/12) were relevant, most patients (9/13) believed that the items were interchangeable and should be combined into 1 item. Patients tended to describe “difficulty focusing” in narrow terms relating to a specific task and described “difficulty thinking clearly” in broader concepts which included confusion, memory, or language retrieval issues.

#### Understanding of PNH-SQ

Participant understanding of instructions and symptom concepts was high, between > 90 and 100% demonstrated understanding. Participant understanding of the response options was very high for each item, from > 80 to 100%. Further, > 80–100% stated that they would be able to easily recall the symptom and its severity within the last 24 h.

### PNH-SQ refinement and finalization

Since participants felt that “difficulty focusing” was interchangeable with “difficulty thinking clearly,” we decided to retain only 1 item. Given that the descriptions provided by participants for “difficulty thinking clearly” aligned more closely with the broader concept of cognitive difficulties and “brain fog,” that item was retained.

The item “sexual difficulties” was also removed. Sexual difficulties among men were primarily experienced as erectile dysfunction (ED) while 1 woman experienced sexual difficulties as low sex drive. While 6 of the 15 participants (1 female, 5 males) reported having experienced sexual difficulties, 13 participants had not experienced this symptom in the past 24 h (Fig. 3). Further, the frequency with which patients experienced sexual difficulty varied. Two male participants reported experiencing ED on an ongoing basis, 1 within the last 24 h and 1 within 5 days of completing the PNH-SQ with the 3 other male participants experiencing ED only in the past (a single instance of ED 2 and 9 years prior for 2 participants). The woman reported experiencing a low sex drive on a weekly basis. In addition, 1 male participant reported that they received clinical confirmation that their ED was not attributable to PNH and the woman reported that the low sex drive was due to her anemia. Due to the limited relevance of the item to women with PNH (1/8), the variable frequency with which it occurred, and the fact that the experience of the symptom could be due to factors external to PNH, the item was dropped. The response options were not modified from the original version as they were well understood and considered appropriate for each item.

Lastly, several minor changes were made to facilitate the e-administration of the measure. The final instrument included questions on 10 symptoms of PNH: abdominal pain, chest discomfort, difficulty sleeping, difficulty swallowing, difficulty thinking clearly, fatigue, headache, muscle weakness, pain in the legs or back, and shortness of breath. The PNH-SQ, intended for daily e-administration, asks the patient to report on the presence/absence of each symptom over a 24-h period and to rate the severity of each symptom they have experienced.

## Discussion

This study describes the development of the PNH-SQ, a content-valid questionnaire developed in accordance with regulatory guidelines [9] and scientific best practices [14]. A literature review of instruments used to assess PNH symptoms in a clinical trial context informed the need for the development of a daily symptom diary that assesses the most salient symptoms of PNH from the perspective of the patient. The PNH-SQ was developed by combining the findings from the literature review, as well as expert clinician advice, and was further refined through patient interviews.

CE interviews with 15 PNH patients confirmed that most patients (i.e., > 90%) experience symptoms due to their PNH and that they experience a wide variety of symptoms [7, 8, 13]. Even while many (7/15) participants were receiving treatment for their PNH, study participants reported experiencing over 27 signs and symptoms due to PNH with the most common being fatigue (87%), abdominal pain (60%), dysphagia (47%), sexual difficulties (40%), and back pain (40%); and one-third (33%) of patients reported cognitive difficulties, headache, muscle weakness, and shortness of breath. A small qualitative study of 29 patients by Weitz et al. [7] reported similarly high rates of fatigue (97%) abdominal pain (59%), dysphagia (41%), and sexual difficulties (47%) [8]. Likewise, similar rates of erectile dysfunction (38%) and fatigue (80%) were reported in the International PNH registry. However, both in the International PNH Registry and in the Weitz study, patients reported substantially higher rates of headache (33% vs. 63% and 76%, respectively) and shortness of breath (33% vs. 64% and 66%, respectively) [7, 8]. Further, participants in our study also reported experiencing leg/back pain (53%), muscle weakness (33%), and sleep problems (27%), which were not reported by the patients in the International PNH Registry or in the qualitative study [7, 8]. Differences in the experience of symptoms may be driven by case mix, including severity of PNH (most patients in our study had self-reported mild disease but severity was not reported in the registry [7] or Weitz study [8]) and treatment status (none of the patients in the Weitz study [8] were treated with eculizumab vs. 25% in the registry [7] and > 45% in our study).

The significance of fatigue in the PNH experience is underscored by the fact that fatigue was the most commonly experienced symptom (13/15; 86.7%) and was considered the most bothersome symptom by the majority (8/15) of patients in this study. The proportion of individuals with PNH experiencing fatigue corresponds with findings from the International PNH Registry and the Weitz study; both of which found that fatigue was the most frequently reported symptom (80% and 97%, respectively) [8]. Moreover, in our study, nearly one-half of participants experienced fatigue in the prior 24-h (7/15) and more than half (4/7) rated the severity as moderate to severe. This is consistent with a cross-sectional survey including 74 participants with PNH in which mean levels of fatigue were severe [15]. Our findings are also consistent with the Weitz study, in which fatigue was reported to occur frequently or almost constantly in 52% of the study participants. Overall, the evidence from this and other studies demonstrate that fatigue is a frequently experienced symptom of PNH.

Fatigue has been positioned as a key secondary endpoint in 2 phase-3 PNH clinical trials [16, 17], both using the FACIT-Fatigue to measure the symptom. Although, the FACIT-Fatigue has been shown to comprehensively capture the fatigue that PNH patients with experience [8], it uses a 7-day recall period. As previously discussed, most patients with PNH experience fatigue frequently and the use of a 7-day recall period does not allow for the measurement of day-to-day variability in fatigue that participants may experience due to changes in disease activity or as a result of the treatment cycle. Although we acknowledge that there is some support for longer recall periods [18], the FDA’s PRO guidance suggests that a shorter recall period is preferable for instruments used in clinical trials due to concerns about recall bias [10]. As has been demonstrated in other chronic diseases, using a 7-day recall period may introduce a recall bias with patients recalling higher symptom intensity than when a daily recall period is used [19, 20]. For example, a study examining fatigue among 97 rheumatology patients confirmed that recall bias was not significant when comparing daily recalls of fatigue to momentary ratings, suggesting that a 24-recall period is suitable for fatigue [21]. The use of a daily symptom questionnaire, such as the PNH-SQ, which can capture day-to-day changes in fatigue and other symptoms can provide a more nuanced representation of the patient’s experience of these symptoms. Additionally, in measuring the daily variability in fatigue, the PNH-SQ may be a more sensitive measure than the FACIT-Fatigue in detecting changes in this symptom.

A widely used PRO measure in PNH clinical trials [16, 17], the EORTC QLQ-C30 was found to inadequately capture symptoms experienced by PNH patients [8]. The study found that while the measure was clear and easy to understand, it contained several items that were of no or low relevance to US patients with PNH, including items such as vomiting, needing help with eating and dressing, nausea, and diarrhea. In our study, none of these signs or symptoms emerged as concepts important to patients with PNH. Importantly, the most common symptoms of PNH such as headache, dysphagia, and abdominal pain reported by participants in that study and in our study are not directly assessed by the EORTC QLQ-C30. In addition, the most commonly experienced and bothersome symptom of PNH, fatigue, is not directly assessed in the EORTC-QLQ-30, which includes a question about “tiredness” rather than fatigue. In fact, the conclusion from the study evaluating the EORTC QLQ-30 as a PRO for patients with PNH was to add items regarding common symptoms of PNH, namely, abdominal pain, headache, and shortness of breath [8]. Evidence from qualitative studies, including our own, suggests that the EORTC QLQ-C30 does not assess all symptoms that are meaningful to patients with PNH [8], an aspect that is critical in the use of PROs in clinical trials [10].

In contrast, to ensure that the items of the PNH-SQ were relevant and comprehensively captured, participants in our study were asked to comment on the relevance of each item. Item relevance on the PNH-SQ was > 50% for all items (range 54.5–100%). Furthermore, when asked about measure comprehensiveness, no patient suggested adding further symptoms. These findings support the content validity and comprehensiveness of the PNH-SQ.

A new PRO instrument—the Quality of Life Questionnaire for patients with Aplastic Anemia and/or Paroxysmal Nocturnal Hemoglobinuria (QLQ-AA/PNH)—was recently developed to measure quality of life in patients with aplastic anemia and PNH [6, 22]. The instrument was developed with significant patient and clinician input, has 54 items with a 14-day recall period for most items and a 6-month recall period for 2 items. While the measure does assess some symptoms, the focus of the items are quality of life impacts and healthcare experience [22]. Indeed, the instrument addresses a significant need for a quality of life tool specific to patients with PNH but it does not comprehensively assess PNH symptoms, their frequency, or their severity. Further, as previously discussed, there is day-to-day variation in experience of PNH symptoms and the 2-week recall period may introduce recall bias in the measurement of symptoms [19–21]. The QLQ-AA/PNH may be a complementary instrument to the PNH-SQ; together these instruments can comprehensively assess both symptom burden and quality of life impact of PNH.

Recently, the patient-reported outcome questionnaire for aplastic anemia and paroxysmal nocturnal hemoglobinuria (PRO-AA/PNH) was developed [23]. However, upon comparison of the measures, only some of the 10 PNH-SQ items are also measured in the PRO AA/PNH (e.g., fatigue, shortness of breath, difficulty concentrating, and dysphagia). For instance, the PRO AA/PNH captures pain in one general question, whilst the PNH-SQ asks about specific issues with pain because this was a significant area of concern raised by patients (items include back, leg, and stomach pain). Additionally, the PRO-PNH/AA captures some signs of PNH (eg, hemoglobinuria, jaundice) and impacts (mood, trouble doing strenuous activities). The PNH-SQ focuses primarily on symptoms of PNH rather than signs or impacts of PNH, and was developed to capture the most relevant symptoms that only patients discussed (e.g., erectile dysfunction was not included because on a day-to-day basis, this was not reported as an issue). Thus, the PNH-SQ may be a more sensitive measure of change in symptoms. Furthermore, the impact of PNH is multi-faceted and arguably warrants a more comprehensive approach to assessment by using something like the QLQ-AA/PNH [6].

The findings from this study must be understood in the context of several limitations. The study included 15 patients with PNH, all of whom were from the US and all interviews were conducted in English which may impact its cross-cultural validity. However, a translatability assessment was conducted to ensure that the instructions, items, and response options could be translated across a variety of different languages, as individuals who speak different languages may express their symptom experience differently. Moreover, in order to obtain a diverse group of participants and best capture the wide range of PNH experiences, recruitment targets were set by age group, ethnicity, race, educational attainment, treatment status, and self-reported disease severity. While there is no evidence to suggest that PNH symptom experience would differ across race or ethnicity, no participants in this study were African American and only 1 Hispanic individual participated in the study. Nevertheless, since saturation was reached, the conduct of additional interviews is unlikely to result in the elicitation of additional, key symptoms that are relevant to patients with PNH.

Further, clinical confirmation of PNH diagnosis was only possible for 4 patients and thus the study relied primarily on self-reported PNH diagnosis for most participants. Considering the nature of the study, the detailed disease-related questions, and the fact that participants were recruited from a PNH or rare disease advocacy organization, it is unlikely that those participants who self-reported their PNH diagnosis did not in fact have PNH. Furthermore, PNH patients with AA were not included in this study; this measure may therefore not comprehensively capture the experience of patients who have both diseases.

In conclusion, the PNH-SQ is a new, content-valid PRO measure suitable for assessing daily symptoms experienced by patients with PNH in a clinical trial context. The development of the PNH-SQ was informed by the literature and by clinician and patient input. While further research is necessary to determine the psychometric properties of the PNH-SQ (i.e., reliability, construct validity, ability to detect change, meaningful change threshold, and scoring algorithm), this study demonstrates that the PNH-SQ is a clear and easy to understand questionnaire that has comprehensive coverage of relevant PNH symptoms. Lastly, because the PNH-SQ was developed for daily administration, it can capture daily variations in symptoms of PNH patients, providing a more granular assessment of symptom changes among patients enrolled in clinical trials.

## Data Availability

The datasets used and analyzed during the current study are available from the corresponding author on reasonable request.

## Abbreviations

AA-MDS: Aplastic Anemia and Myedolysplastic Syndromes International FOUNDATION
CD: Cognitive debriefing
CE: Concept elicitation
ED: Erectile dysfunction
EORTC QLQ-C30: European Organisation for Research and Treatment of Cancer Quality of Life of Cancer Patients
FACIT-Fatigue: Functional assessment of chronic illness therapy-fatigue
FDA: Food and drug administration
PNH: Paroxysmal nocturnal hemoglobinuria
PNH-SQ: PNH symptom questionnaire
PRO: Patient-reported outcomes
PRO-AA/PNH: Patient-reported outcome questionnaire aplastic anemia and paroxysmal nocturnal hemoglobinuria
RPV: Rare patient voice
QLQ-AA/PNH: Quality of life questionnaire for patients with aplastic anemia and/or paroxysmal nocturnal hemoglobinuria
